# Cadmium Transporters in the Kidney and Cadmium-Induced Nephrotoxicity

**DOI:** 10.3390/ijms16011484

**Published:** 2015-01-09

**Authors:** Hong Yang, Yan Shu

**Affiliations:** Department of Pharmaceutical Sciences, School of Pharmacy, University of Maryland at Baltimore, Baltimore, MD 21201, USA; E-Mail: hong.yang@umaryland.edu

**Keywords:** cadmium, transporter, nephrotoxicity

## Abstract

Among the organs in which the environmental pollutant cadmium causes toxicity, the kidney has gained the most attention in recent years. Numerous studies have sought to unravel the exact pathways by which cadmium enters the renal epithelial cells and the mechanisms by which it causes toxicity in the kidney. The purpose of this review is to present the progress made on the mechanisms of cadmium transport in the kidney and the role of transporter proteins in cadmium-induced nephrotoxicity.

## 1. Introduction

**Figure 1:**
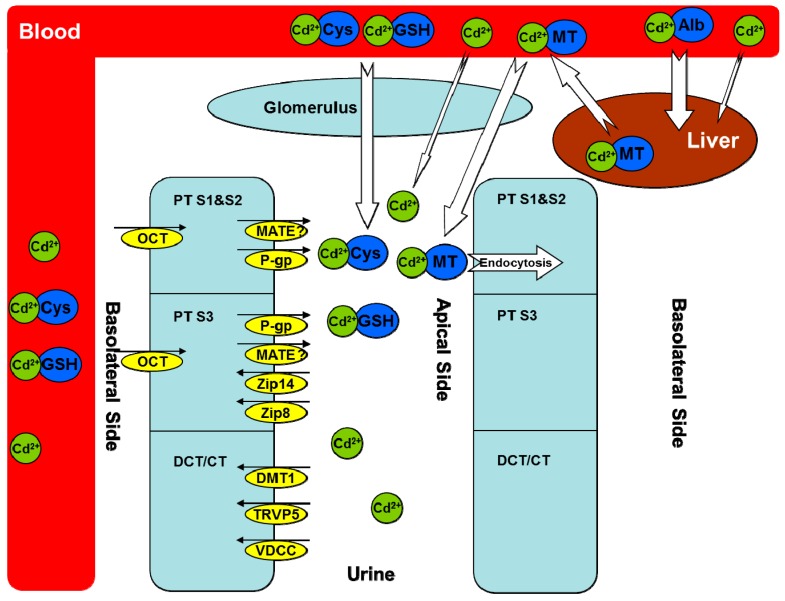
Cadmium transporters. Cadmium (Cd) is mostly bound to albumin in the circulation after absorption and then taken up by the liver. Some cadmium is bound to thiol-containing groups such as glutathione (GSH) and l-cysteine (Cys), which may be absorbed through both basolateral and apical sides of renal proximal tubular (PT) cells. In the liver, cadmium stimulates the synthesis of metallothioneins (MTs) and forms the Cd–MT complex to be released into the circulation. Cd–MT is easily filtered through the glomerulus and reabsorbed into PT segment 1 (S1) and segment 2 (S2) via endocytosis. Multiple transporters are responsible for the transport of free cadmium ions in different PT segments, distal convolute tubular, and connected tubular cells, either from the blood to the epithelial cells or from the epithelial cells to the urine. Alb, albumin; MT, metallothionein; GSH, glutathione; Cys, l-cysteine; PT, proximal tubular; DCT, distal convoluted tubular; CT, connected tubular; Cd^2+^, cadmium ion; OCT, organic cation transporter; MATE, multidrug and toxin extrusion proteins; P-gp, P-glycoprotein; Zip 8, zinc/iron-regulated transporter 8; Zip 14, zinc/iron-regulated transporter 14; DMT1, divalent metal-ion transporter-1; TRVP5, transient receptor potential vanilloid type 5; VDCC, voltage-dependent calcium channels. The small black arrows indicate the direction of Cd transporters in the kidney. Our unpublished data [4] suggest a role of MATE in Cd transport, which needs to be further characterized (indicated by a question marker). The white arrows indicate the direction of Cd transport between blood and either renal glomerulus or hepatocytes, with the size showing the relative contribution from different Cd complexes.

The issue of cadmium pollution is accompanied by human industrial civilization. Every year, thousands of tons of cadmium-containing pollutants are discarded into our environment, which poses an increasing health risk to our food and drinking water. It is now well accepted that cadmium can accumulate in many organs, including liver, kidney, pancreas, and testis, and adversely affect the functions of these organs. Among them, the kidney is recognized as a major target of cadmium-induced toxicity. Clearly, knowledge of cadmium transport pathways will allow for a better understanding of cadmium-induced nephrotoxicity. After decades of research, a variety of transport proteins have been characterized that play a role in renal cadmium accumulation. These include metallothioneins (MTs), zinc transporters, calcium transporters, divalent metal-ion transporter-1, and some recently identified ones, such as organic cation transporters, multidrug and toxin extrusion proteins, and additional cadmium-binding proteins containing thiol groups. Several mechanisms have also been proposed to account for cadmium-induced nephrotoxicity, including oxidative stress, cell apoptosis, and glomerular contraction, which have been well reviewed in the literature [1,2,3]. The aim of this review is to summarize the progress made on renal cadmium transporting pathways (Figure 1) and their role in cadmium-induced nephrotoxicity.

## 2. Metallothioneins (MTs)

### 2.1. Evidence as a Cadmium Transporter

The family of MTs is a group of cysteine-rich proteins with molecular weights (MW) ranging between 6 and 7 kDa [5]. Since its discovery from cadmium-binding proteins in horse renal cortex [6], the role of MTs in the transport of cadmium has undergone comprehensive investigation. Due to the abundant thiol groups in their cysteine residues, having high affinity to metal ions, MTs are thought to play an important role not only in the homeostasis of physiological metal ions, such as zinc, copper, and selenium, but also in protection from the toxicity of heavy metals, such as cadmium, mercury, lead, and arsenic.

After chronic exposure of rodents with cadmium chloride, most cadmium molecules are bound to albumin in the circulation system and the metal-protein complex is readily and mainly taken up by the liver (Figure 1). In the liver, cadmium acts as a stimulus to MT synthesis and is bound to MTs as a protective mechanism for the body to sequester xenobiotic cadmium. Cadmium–metallothionein (Cd–MT) complex is then released to the circulation system; due to the small size of MTs, the complex is easily filtered through the glomerulus and reabsorbed by the proximal tubular (PT) epithelial cells via endocytosis [7,8]. There is direct evidence of cadmium relocation from the liver to the kidney. In a study by Chan and colleagues [9], rats were treated with cadmium chloride (3 mg/kg/day) for two weeks and the livers of cadmium-treated rats were transplanted to the control group. The levels of cadmium and MTs in the kidney of the transplanted rats increased significantly, while those in the liver decreased in a time-dependent manner. A clear role of MTs in renal cadmium accumulation has been demonstrated by Liu and colleagues [10] who showed that the cadmium concentration in the kidney of MT-I/II knockout mice was only one-fifth that of wild-type mice after treatment with cadmium chloride for six months. Likewise, Dorian and colleagues [11] have reported that over eighty percent of Cd–MT complexes reside in the kidney after fifteen minutes following intravenous injection; consistent with pathological findings, the complex was mainly taken up into the segment 1 (S1) and segment 2 (S2) of the proximal tubules. The Cd–MT complex has been reported to be rapidly degraded after entry into the epithelial cells [5]. However, the released cadmium is then bound to newly synthesized MTs in the cytoplasm. The binding of cadmium to MTs is a detoxification mechanism that is saturable at high concentrations of cadmium for which subsequent renal toxicity may become apparent.

### 2.2. Role in Cadmium-Induced Nephrotoxicity

As reviewed above, MTs play a critical role in cadmium accumulation in the kidney. It seems that an enhanced activity of MTs contributes to cadmium-induced nephrotoxicity. However, this concept was disproved by Liu *et al.* [12], whose study showed that although cadmium accumulation in the kidney of MT-I/II knockout mice was no more than ten percent that of the wide-type controls, the knockout mice were surprisingly more susceptible to the cadmium-induced nephrotoxicity. It was estimated that the maximum tolerable cadmium dose in the knockout mice group was only one-eighth of the controls. The authors concluded that cadmium concentration was much lower in the kidney of MT-I/II knockout mice, however, cadmium was directly exposed to the biological molecules and the organelles in the renal epithelia cells without the protection from MTs [10]. In the wide type mice, contrastingly, the renal damage only became evident at very high amounts of cadmium in excess of the binding ability of MTs in the cell. Nowadays, MTs have been acknowledged as protective agents rather than the culprit in cadmium-induced nephrotoxicity.

## 3. Zinc Transporters

### 3.1. Evidence as a Cadmium Transporter

Cadmium is a non-essential toxic heavy metal, which may explain why there is no specific cadmium transporter system in the body. However, it is well established that cadmium competes with other metals for transporter-mediated entry into the cell. Among these transporters, those for zinc, which is the congener of cadmium and a physiological essential metal ion, gain the most attention.

ZRT/IRT-Related Proteins (ZRT, zinc-regulated transporters; IRT, iron-regulated transporters), also called ZIP proteins, and firstly identified from the root of iron-deficient plants, have recently been shown to play a pivotal role in zinc transport across the cellular membrane in the intestine and proximal tubules [13] (Figure 1). By utilizing mouse proximal tubular cells, Fujishiro and colleagues [14] have demonstrated that knockdown of ZIP8 and ZIP14, which were highly expressed in the cell line, resulted in significantly reduced cadmium uptake on the apical side. The knockdown of ZIP8 was also found to be protective against cadmium toxicity by reducing its uptake in a ZIP8-transfected HEK cell line [15]. In addition, the role of ZIP8 and ZIP14 in cadmium transport has been tested in rat basophilic leukemia (RBL-2H3) cells [16,17]. While both ZIP8 and ZIP14 were highly expressed in the RBL-2H3 cells, ZIP8 seemed to play a more crucial role since the uptake of cadmium was increased following knockdown of ZIP14 by siRNA, rather than significantly reduced. The study by Barbier and colleagues [18] may give a better insight into the function of zinc transporters on cadmium transport under physiological conditions. By using the nephron microinjection technique, they showed that cadmium uptake could be inhibited by nearly thirty percent in distal convoluted tubules (DCT) by co-injection with a small amount of zinc ion, but no inhibition was observed in proximal tubules (PT). Although zinc transporters are expressed in both DCT and PT, cadmium may have a higher affinity for other transporters in PT segments, the uptake of which might not be inhibited by zinc ions. The role of zinc transporters in cadmium uptake has been well established from *in vitro* studies; however, further *in vivo* studies are needed.

### 3.2. Role in Cadmium-Induced Nephrotoxicity

Zinc transporters expressed on the apical side of renal epithelia cells are responsible for reabsorbing most of the zinc ions. Findings from *in vitro* studies suggested a role of zinc transporters in renal cadmium accumulation and toxicity. However, persuading evidence from *in vivo* characterization is lacking. An earlier study performed by Tang *et al.* [19] found that pre-treatment with zinc ions attenuated renal cadmium accumulation and reduced nephrotoxicity, possibly by displacing cadmium from the Cd–MT complex. Dietary co-administration of cadmium chloride with zinc was found to significantly reduce renal cadmium accumulation as compared with the cadmium-only group [20]. However, the possible effect of zinc as an inhibitor of intestinal cadmium absorption could not be ruled out. As a matter of fact, the results from another study showed that although co-injection of cadmium chloride and zinc chloride could significantly reduce cadmium-induced nephrotoxicity, renal cadmium accumulation was unaltered [21]. Data from clinical studies are not conclusive regarding the role of zinc transporters in cadmium-induced nephrotoxicity; the genetic polymorphisms in *Slc39a8* gene (encoding for ZIP8) and *Slc39a14* gene (encoding for ZIP14) were found to likely affect cadmium blood and urine concentrations in humans [22], although the specific functional changes associated with the polymorphisms need further validation. In a recent clinical study, it was reported that low levels of serum zinc was associated with an increased risk of cadmium nephrotoxicity, however, the role of zinc transporters was not determined [23]. Further *in vivo* studies are warranted to characterize the role of zinc transporters in renal accumulation of cadmium and its induced nephrotoxicity. For example, studies in ZIP knockout mice, particularly the tissue-specific knockout mice, may provide us with a more definitive conclusion.

## 4. Calcium Transporters

### 4.1. Evidence as a Cadmium Transporter

Another transporting route for cadmium is the calcium transporter, which is also an essential transporter for life. Wang and colleagues [24] found that increased calcium in the tubular luminal could decrease cadmium accumulation in rabbit tubular cells, suggesting the role of calcium transporters in renal cadmium uptake. The superfamily of transient receptor potential (TRP) channels is widely expressed in many tissues such as intestine, kidney, and placenta, and these channels are established as the major pathways for calcium absorption and reabsorption [25]. Transient receptor potential vanilloid type 6 (TRPV6) and transient receptor potential vanilloid type 5 (TRPV5) are two major members in this superfamily, with TRPV6 mainly expressed in duodenum and placenta. TRPV5 is expressed in the distal convoluted tubules and the connected tubules (Figure 1). The roles of TRPV6 and TRPV5 in cadmium transport have been characterized in recent years. Kovacs *et al.* [26] reported that the uptake of both calcium and cadmium increased significantly in the HEK293 cells over-expressing human TRPV6 as compared to the parent cell line, and that the uptake of calcium could be efficiently inhibited by cadmium, suggesting competition between the two types of ions for the same transport system. Moreover, cadmium accumulation mediated by TRPV6 could be inhibited by the non-specific human TRPV6 inhibitor 2-aminoethoxydiphenyl borate (2-APB). In addition, overexpression of human TRPV5 was found to increase the uptake of cadmium and sensitize HEK293 cells to cadmium-induced toxicity [25].

The role of another family of calcium channels, voltage-dependent calcium channels (VDCC), in cadmium transport has also been explored recently [27]. Although the data suggested little function of VDCC in cadmium transport, it was not totally ruled out since additional potent cadmium transporters could dominate cadmium transport across the membrane of the studied cells.

### 4.2. Role in Cadmium-Induced Nephrotoxicity

Little evidence has been reported so far regarding the role of specific calcium transporters in cadmium-induced nephrotoxicity. Results from *in vitro* studies suggested that calcium transporters are not major transporters for cadmium uptake in renal epithelia cells, and that their function may be well complemented by other high affinity and/or high capacity cadmium transporters [8,27]. While this is probably an explanation for the lack of evidence for calcium transporters in cadmium-induced nephrotoxicity, further work is needed to illustrate the physiological importance of calcium transporters on cadmium accumulation and toxicity, particularly from carefully designed *in vivo* studies.

## 5. Divalent Metal-Ion Transporter-1 (DMT1)

### 5.1. Evidence as a Cadmium Transporter

Divalent metal-ion transporter-1 (DMT1), a hydrogen-coupled metal iron transporter, not only plays a crucial role in iron homeostasis, but also can mediate transport of essential and toxic divalent metal ions, such as Zn^2+^, Mn^2+^, Co^2+^, Pb^2+^, Cd^2+^, and Cu^2+^. Due to its wide localization in proximal tubule S3 segment, distal convoluted tubule, and connected ducts (Figure 1), the role of DMT1 in renal accumulation of cadmium has been examined in recent years. By using the microinjection technique, Barbier *et al.* found that DMT1 might play a crucial role in cadmium absorption in distal tubule cells [28]. A significant decrease in the expression of DMT1 has been observed in cadmium-resistant mouse embryonic cells [29] and DMT1 even had a higher affinity for Cd^2+^ than Fe^2+^ as reported by Illing and colleagues [30]. In addition, it has been reported that cadmium accumulation in mouse proximal tubule cells decreased nearly thirty percent following DMT1 knockdown by siRNA [14]. This result is consistent with that obtained from Caco-2 cells, in which the knockdown of DMT1 caused an over fifty percent decrease in cellular cadmium accumulation [28].

### 5.2. Role in Cadmium-Induced Nephrotoxicity

As reviewed above, the role of DMT1 in cadmium transport has been well established in *in vitro* cellular models. However, its contribution to renal accumulation of cadmium and cadmium-induced nephrotoxicity has not been rigorously verified in the more complex *in vivo* systems. While we await more cogent evidence, several studies have provided some clues. In an early research work, chronic administration of cadmium was found to result in renal anemia in rats. Although the iron concentration was significantly lower in the plasma of cadmium-treated group, no significant difference was found in erythropoietin (EPO) production [31]. It is possible that the anemia was due to decrease in iron reabsorption as a result of decreased DMT function in the kidney after cadmium treatment. Likewise, results of Kim *et al.* revealed that an iron-sufficient diet could decrease cadmium accumulation in the kidney but not in the liver of wild-type mice, suggesting that iron could reduce cadmium absorption in the kidney possibly via competitive inhibition of DMT1 [32]. In the future, the role of DMT1 in cadmium-induced nephrotoxicity should be characterized *in vivo*.

## 6. Organic Cation Transporters (OCTs) and Multidrug and Toxin Extrusion Proteins (MATEs)

### 6.1. Evidence as a Cadmium Transporter

Organic cation transporters (OCTs) and multidrug and toxin extrusion proteins (MATEs) work in concert in the elimination of cationic drugs and toxins from the kidney (Figure 1). Human OCT2, highly expressed in the basolateral membrane of proximal tubular cells, and MATE1/2-K, expressed in the apical membrane, play a crucial role in the elimination of a wide range of exogenous and endogenous molecules [33]. The evidence for the involvement of OCTs in cadmium transport became apparent recently. Soodvilai *et al.* [34] reported that overexpression of either rabbit OCT1 or OCT2 increases the intracellular accumulation of cadmium in Chinese hamster ovary (CHO-K1) cells, and the effect could be inhibited significantly by the OCT substrate tetraethylammonium (TEA). In order to examine the role of OCTs *in vivo*, they used a bilateral ureteral ligation rat model to examine the basolateral uptake of cadmium independently from glomerular filtration and tubular absorption. Consistent with their *in vitro* experiments, cadmium accumulation in the kidney was reduced by over 80 percent when co-treated with TEA in this model. MATEs might also be cadmium transporters in proximal tubular epithelial cells considering the wide overlap of substrates between OCTs and MATEs. The basolateral OCTs and the apical MATEs may work collaboratively as a renal detoxification mechanism to eliminate cadmium. Data from our lab showed that cadmium uptake significantly increased in human MATE1 over-expression HEK293 stable cells as compared to the mock controls [4].

### 6.2. Role in Cadmium-Induced Nephrotoxicity

Since the majority of transporters we reviewed above seem to be responsible for cadmium accumulation in the kidney, the organic cation transport system consisting of OCTs and MATEs is particularly attractive for its potential ability to eliminate cadmium and attenuate not only renal toxicity but also systemic exposure of cadmium. By using the genetic mouse models deficient of respective OCT and MATE function that are currently available, we can test the role of these transporters in acute and chronic cadmium accumulation and nephrotoxicity. However, it should be noted that only approximately 0.7 percent of the total cadmium dose has been found to accumulate in the kidney one hour after cadmium injection [34]. The actual contribution of OCTs and MATEs to cadmium accumulation and elimination in the kidney should be analyzed with caution.

## 7. Other Transport Mechanisms and Transporters

Other thiol-containing groups in the plasma such as glutathione (GSH), l-cysteine (Cys), l-homocysteine (Hcy), and *N*-acetyl-l-cysteine (NAC) may also participate in cadmium transport. Due to its high affinity for thiol moieties, cadmium will form complexes with these amino acids and peptides (Figure 1). Results from Zalup *et al.* showed that co-administration of inorganic cadmium with GSH and Cys increased renal cadmium accumulation in rats, and the accumulation was both basolaterally and apically dependent [35]. Wang and colleagues [24] found that a Cys–Cadmium–Cys complex could be freely filtered through glomerulus and then reabsorbed through the amino acid transporters in the tubular apical membrane. Contrastingly, GSH has been found to be able to reduce renal cadmium accumulation and attenuate renal toxicity after Cd–MT injection [19,36]. GSH might function by displacing cadmium from MTs, and the GSH–Cd complex is not as easily reabsorbed as Cd–MT in renal epithelia cells.

Other transporters may participate in cadmium transport as well. *P*-Glycoprotein (*P*-gp), a member of the ATP-binding cassette (ABC) transporter superfamily usually located on the apical side of the epithelial membrane, is responsible for efflux of a wide range of structurally diverse compounds (Figure 1). The role of *P*-gp in cadmium transport was examined in kidney-derived cell lines [37,38]. *P*-gp was found to only account for a small portion (nearly 10 percent) of cadmium efflux in the pig kidney epithelial cells (LLC-PK1), and this was increased to nearly 20 percent by *P*-pg over-expression in LLC-PK1 cells [37]. These findings are consistent with the results from another study which found that the *P*-gp inhibitor and monoclonal antibody could reduce cadmium efflux in immortalized cells derived from the S1 segment of rat kidney proximal tubules [38]. Likewise, Martin *et al.* [39] reported that manganese was a more efficient protective agent than zinc against cadmium toxicity and, as a result, they hypothesized that the manganese transport system may be a contributor to cadmium transport as well.

## 8. Conclusions

It is well recognized that MTs play a pivotal role in cadmium disposition after chronic exposure to cadmium. After synthesis and release from the liver, the Cd–MT complex is readily filtered through the glomerulus and reabsorbed in proximal tubular S1 and S2 segments. However, cadmium accumulation has been found across the renal cortex and medulla. In particular, although the renal elimination rate of cadmium is quite slow, it seems that transporter proteins are critically involved in the process. After decades of research, various transporting systems have been identified to play a role in renal cadmium accumulation. Nevertheless, it should be emphasized that the evidence regarding the involvement of certain newly identified transporters in cadmium transport has been mainly collected from *in vitro* studies; hence, well-designed *in vivo* studies are needed to further confirm and characterize the contribution of individual transport systems to the overall renal cadmium transport and cadmium-induced nephrotoxicity under different conditions.
